# Spatial and temporal patterns of indicators of climate change and variability in the Arab world in the past four decades

**DOI:** 10.1038/s41598-023-42499-y

**Published:** 2023-09-13

**Authors:** Salahuddin M. Jaber, Mahmoud M. Abu-Allaban, Raja Sengupta

**Affiliations:** 1 Department of Geography, McGill University, Montreal, QC H3A0B9 Canada; 2 Department of Water Management and Environment, Prince El-Hassan bin Talal Faculty for Natural Resources and Environment, The Hashemite University, P.O. Box 330127, Zarqa, 13133 Jordan; 3 Department of Geography, Bieler School of Environment, McGill University, Montreal, QC H3A0B9 Canada

**Keywords:** Climate sciences, Environmental sciences

## Abstract

A comprehensive assessment of the spatial and temporal patterns of the most common indicators of climate change and variability in the Arab world in the past four decades was carried out. Monthly maximum and minimum air temperature and precipitation amount data for the period 1980–2018 were obtained from the CHELSA project with a resolution of 1 km^2^, which is suitable for detecting local geographic variations in climatic patterns. This data was analyzed using a seasonal-Kendall metric, followed by Sen’s slope analysis. The findings indicate that almost all areas of the Arab world are getting hotter. Maximum air temperatures increased by magnitudes varying from 0.027 to 0.714 °C/decade with a mean of 0.318 °C/decade while minimum air temperatures increased by magnitudes varying from 0.030 to 0.800 °C/decade with a mean of 0.356 °C/decade. Most of the Arab world did not exhibit clear increasing or decreasing precipitation trends. The remaining areas showed either decreasing or increasing precipitation trends. Decreasing trends varied from −0.001 to −1.825 kg m^−2^/decade with a mean of −0.163 kg m^−2^/decade, while increasing trends varied from 0.001 to 4.286 kg m^−2^/decade with a mean of 0.366 kg m^−2^/decade. We also analyzed country-wise data and identified areas of most vulnerability in the Arab world.

## Introduction

Global warming is a widely accepted reality, as evidenced by a plethora of articles that have reported a worldwide rise in air temperature in the twentieth century^1,2^. Further, this rise has intensified and shown unprecedented increasing rates in the current century. Temperature rise, which is largely blamed on greenhouse gases; mainly carbon dioxide and methane^3^, has many devastating impacts on natural resources, freshwater availability, drought, and agriculture^4–6^.

Implications of temperature rise include heat waves^7,8^, windstorms^9^, dust storms^10^, storm surges^11^, drought^12^, flooding^13^, food insecurity^14^, food and water borne diseases^15,16^, and vector borne diseases^17^. The frequency and severity of these implications vary spatially and temporally^18–21^. In addition to these consequences, the Arab world is particularly vulnerable to political instability and violence that might escalate to armed conflicts if drought conditions put extra pressure on the already depleted freshwater resources and constrains food production^22,23^.

The Arab world, which involves twenty-one countries located in northern Africa and western Asia, in addition to Comoros in the Indian Ocean, is one of the driest regions in the world. It spans over a vast area of arid to semiarid territories that receive less than 50 mm of annual precipitation^24,25^. Low precipitation and high evaporation rates result in limited freshwater resources and dry soils that cannot sustain large scale agriculture, and thereby fail to fulfill basic needs of food and drinking water. For example, Somalia has suffered from frequent harsh droughts that caused famines^26^. Higher air temperature and lower precipitation are likely to bring more heatwaves and drought that would jeopardize the fragile food security in the region. Additionally, other parts of the Arab world (e.g., Oman) face the threat of receiving intensified tropical storms like Cyclone Gonu of 2009, which was the strongest tropical cyclone over the Arabian Sea since 1970 and resulted in enormous economic losses^27^.

According to the fifth Intergovernmental Panel on Climate Change (IPCC) report, the Arab world is vulnerable to deleterious consequences of climate change, including precipitation reduction and air temperature rise^3^. Ozturk et al. (2021)^28^ investigated projected changes in climate extreme indices over Middle East and North Africa (MENA) by analyzing changes in the daily maximum and minimum temperature and precipitation for the end of the twenty-first century using two regional climate models downscaled to 50 km resolution and reported an intensification of temperature- and precipitation- based extreme indices. Waha et al. (2017)^25^ argued that the MENA region could be heavily challenged by severe consequences of climate change such as rising food and water demand due to a large population, which is projected to double by 2070^29^. Similar findings are confirmed by Namdar et al. (2021)^30^, who point out that countries like Yemen, Djibouti, Syria, Oman, Iraq, and Libya are more vulnerable to climate change than the rest of the Arab world.

In the recent past, the Arab world region and other neighboring Mediterranean countries have already suffered severe weather events such as the harsh summer of 2007, which was associated with widespread wildfires throughout Greece^31,32^. Cornforth et al. (2017)^33^ asserted that several countries in the Arab world are getting hotter and drier and, if no action is taken to reduce global emissions of greenhouse gasses, the region is expected to experience an average increase in temperature of 2.3 °C and a decrease in precipitation of up to 20% by the end of this century. Zittis et al. (2022)^10^ warned of a faster warming rate in the Eastern Mediterranean and the Middle East, where air temperature will rise two times faster than the global mean, and precipitation will decline at unprecedented rates. Cook and colleagues analyzed 900 years (1100–2012) of Mediterranean drought variability in the Old-World Drought Atlas^5^ and came up with the conclusion that the recent fifteen-year drought (1998–2012) in the Levant is the driest on record, with an 89% likelihood that this drought is drier than any comparable period of the last 900 years^34^.

Hamdi et al. (2009)^35^ examined data from six meteorological stations distributed around Jordan to detect trends indicative of climate change in the region, and noted strong trends indicating that annual minimum temperature has increased in the last decade of the twentieth century. Abu Sada et al. (2015)^36^ considered data from several meteorological stations located in northern Jordan, southern Syria, and north-eastern Saudi Arabia between 1980 and 2010, and noted a 0.2 to 0.6 °C/decade increase in the rate of air temperature, along with a 5 to 26 mm/decade decrease in the rate of annual precipitation. Subyani et al. (2016)^37^ investigated characteristics of the observed daily rainfall series at six stations near Jeddah, Saudi Arabia, over the period of 1971–2012, and came up with the conclusion that rainfall is getting more intense in the rainy months (October–April) while the dry months (May–September) are getting drier.

Schilling et al. (2020)^38^ studied and compared climate change vulnerability of Arab countries in north Africa, including Algeria, Egypt, Libya, Morocco, and Tunisia, and reported strong air temperature increases and a high drought risk for these countries. Trend analysis of minimum and maximum air temperature from 30 meteorological stations distributed throughout Morocco revealed significant increasing trends in warm temperature events and decreasing trends in cold extremes during the period from 1960 to 2016^39^. Similar results are reported from Sudan^40^ and Mauritania^28^.

However, most of these studies were based either on data collected from sparsely located and unevenly distributed meteorological stations with paucity of historical data [e.g., 35–40], or regional modelling with low spatial resolution (e.g.^7^). The current work complements previous studies by conducting a comprehensive assessment of the regional manifestations of global warming in the Arab world. This assessment was conducted using processed climate data made available by the Climatologies at High Resolution for the Earth's Land Surface Areas (CHELSA) version 2.1 dataset^41^. Hosted by the Swiss Federal Institute for Forest, Snow, and Landscape Research WSL, CHELSA project offers spatially continuous gridded air temperature and precipitation data for earth’s surface starting as early as 1979. CHELSA data is generated from a statistical downscaling of global ERA5 reanalysis to an unprecedented high resolution of 30 arc sec (⁓1 km), which is suitable for detecting relatively local geographic variations in climatic patterns. The ERA5 is the latest generation of the European Center for Medium-Range Weather Forecasts (ECMWF) reanalysis for the global climate and weather for the past eight decades. CHELSA air temperature data is more reliable than the previously used interpolation based on climate station data, especially in mountainous regions and in areas with low station density. Additionally, predictions of CHELSA precipitation patterns are more accurate than previous interpolations^41^.

CHELSA data have been used in a variety of applications, including mountain biodiversity^42^, composition of plant communities^43^, insect abundance^44^, and climate zonation^45^. To the best of our knowledge, this study is the first to use CHELSA high-resolution data for obtaining information about the spatial and temporal trends of air temperature and precipitation patterns, the two most common indicators of climate and its variability and change in any region. Our analysis spanned the past 39 years (1980–2018) and covered 21 contiguous Arab countries, in addition to the Western Sahara.

## Study area

**Figure 1:**
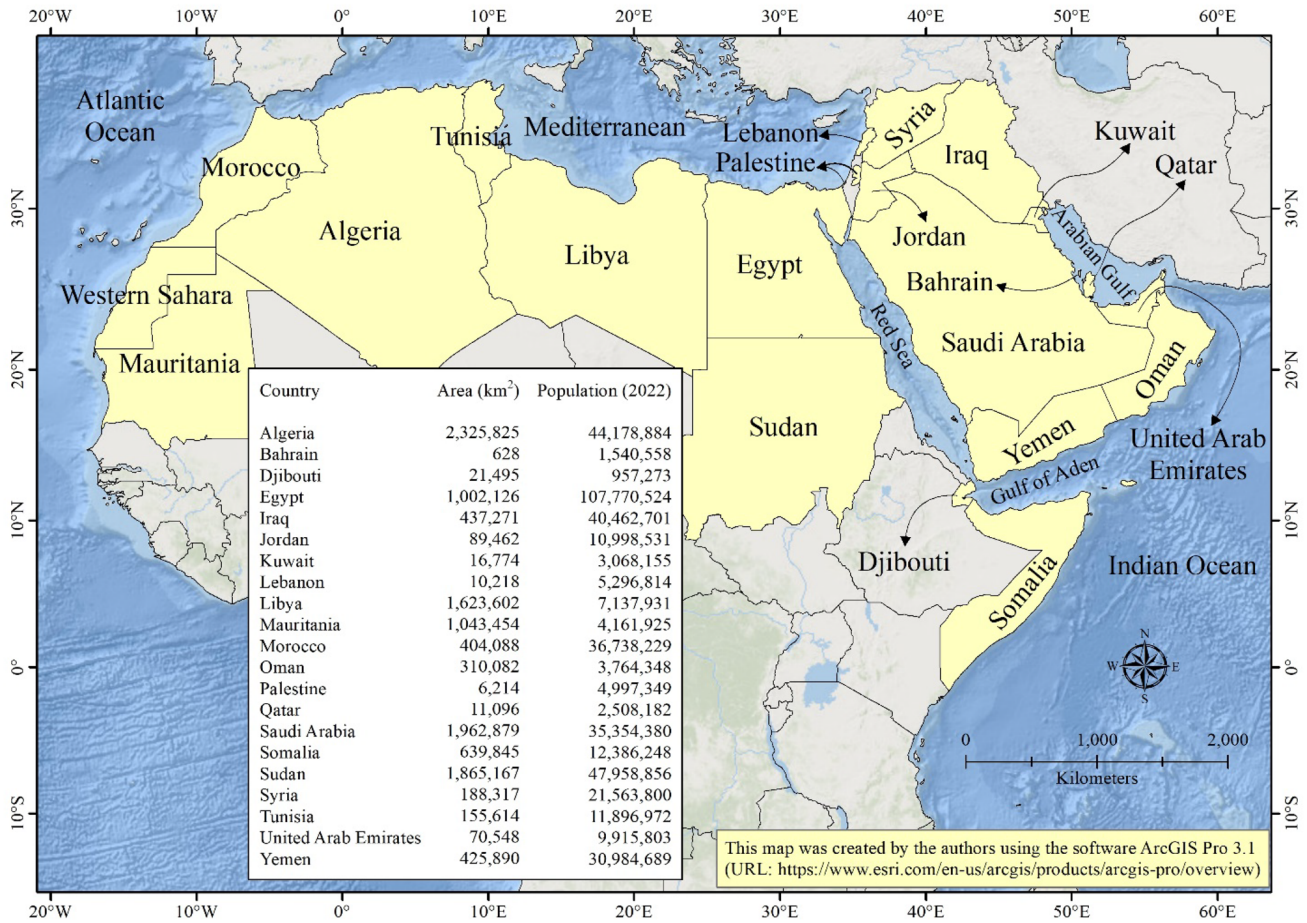
Location map of the Arab countries. Included is a list of the areas and populations of the Arab countries.

The geographic scope of the research (Fig. 1) covers twenty-one adjacent countries within the Arab region, spanning across northern Africa and western Asia. This region also includes the geopolitically disputed Western Sahara region in western Africa. Furthermore, in western Asia, Palestine is comprised of two distinct areas: the Gaza Strip and the West Bank (http://www.leagueofarabstates.net/ar/aboutlas/Pages/CountryData.aspx).

Encompassing an area of approximately 12.9 million km^2^, the study area is home to a population exceeding 444.2 million people. Algeria stands as the largest Arab nation, while Bahrain is the smallest. Egypt is the most populous Arab country, with Djibouti having the least population. Despite shared historical, cultural, linguistic, and religious ties, the Arab countries exhibit significant demographic, social, and economic disparities. Urban populations are concentrated around major urban centers, including capital cities. Only the Gulf Cooperation Council (GCC) countries are classified as high-income economies. Jordan, Iraq, and Libya fall into the upper-middle-income category. Somalia, Sudan, Syria, and Yemen are considered low-income economies, while the remaining Arab countries are classified as lower-middle-income economies.

The study area exhibits a remarkable variation in its topography, ranging from −412 m below mean sea level in the eastern region (at the Dead Sea on the border between Jordan and Palestine) to an elevation of 3957 m above mean sea level in the western area (at Toubkal Peak in the Atlas Mountains of Morocco). This diversity includes prominent mountain ranges such as the Atlas Mountains in northwestern Africa, the Hoggar Mountains in the central Sahara, the Somali Mountains in the northern Horn of Africa, the Yemen-Hadhramout-Dhufar and Hijaz-Asir ranges in the Arabian Peninsula’s southern, southwestern, and western parts, the Lebanon Mountains and Zagros-Taurus ranges in the northern Levant, and the Oman Mountains in the eastern Arabian Peninsula. Noteworthy are also the mountain ranges along the western Red Sea shores in Egypt and Sudan, as well as those in the Sinai Peninsula, southwestern Sudan, and southern Libya.

Approximately 97% of the Arab world falls within the arid climate zone, while the remaining 3% occupies the warm temperate climate zone. This warm temperate climate is primarily found in the Atlas Mountains of northwestern Africa and the fertile crescent region in the northern Levant. This climatic variability follows a gradual trend from lower to higher latitudes, with temperatures and precipitation increasing in tandem.

The study area’s landscape is predominantly composed of barren lands (82%), followed by shrublands/grasslands (14%), and then croplands (3%). Forests, savannas, and wetlands cover less than 1% of the total area. The region is characterized by several extensive deserts, including the Sahara Desert spanning much of North Africa, the Sinai Desert on the Sinai Peninsula, the Syrian Desert stretching northward from the Arabian Peninsula, and the Arabian Desert encompassing the majority of the Arabian Peninsula. Shrublands and grasslands are noticeable as wide belts stretching from west to east in the northern and southern regions of the Arab world. Croplands are primarily concentrated in the Atlas Mountains of northwestern Africa, along the Nile River and its delta, and in the fertile crescent of northern Levant, with sparse areas in southern Sudan. Patches of savannas can be found in the northern parts of the Atlas Mountains, along the Mediterranean coast, and in the fertile crescent region, as well as parts of Sudan and Somalia. Natural forests are mainly located in mountainous regions and flood plains, while limited wetlands are scattered across Mauritania, Algeria, Tunisia, Egypt, Yemen, and Iraq.

## Data and methods

In the present study, global maximum and minimum 2-m air temperature (in Kelvin/10) as well as the global monthly precipitation amount (in kg.m^-2^/100) data for the period 1980–2018 were downloaded from the CHELSA website (https://chelsa-climate.org/downloads/). A total of 1404 files representing 1404 months (39 years × 12 months × 3 variables) were obtained with a Geographic Coordinate System (GCS) referenced to the World Geodetic System 1984 (WGS84) horizontal datum in GeoTIFF format. For maximum and minimum air temperature data, every file representing one month was multiplied by 0.1, converted to degrees Celsius by subtracting 273.15, projected to the world Mollweide coordinate system, and clipped according to the political borders of the Arab world. For precipitation data, every file representing one month was multiplied by 0.01, projected to the world Mollweide coordinate system, and clipped according to the political borders of the Arab world.

Seasonal-Kendall test available in the ‘Generate Trend Raster’ tool in the geographic information system (GIS) ArcGIS Pro 3.1 (https://pro.arcgis.com/en/pro-app/3.0/tool-reference/image-analyst/generate-trend-raster.htm) was applied. The goal was to determine whether there are monotonic trends in the three space–time variables maximum and minimum air temperature and precipitation amount in the Arab world in the period 1980–2018. The tool accepts multidimensional raster data as input. Hence, for each of the three variables, a multidimensional Cloud Raster Format (CRF) dataset was formed as follows. (1) A new multidimensional mosaic dataset was created using the ‘Create Mosaic Dataset’ tool. (2) Time-series rasters (i.e., monthly maximum air temperature or monthly minimum air temperature or monthly precipitation amount) were added to the multidimensional mosaic dataset using the ‘Add Rasters to Mosaic Dataset’ tool. (3) Multidimensional metadata information was added to the multidimensional mosaic dataset using the ‘Build Multidimensional Info’ tool. (4) Finally, the multidimensional mosaic dataset was converted into multidimensional CRF using the ‘Copy Raster’ tool. Each of the three multidimensional CRF datasets that were created was used as input for the ‘Generate Trend Raster’ tool. The output from applying seasonal-Kendall test is a multidimensional CRF dataset, which comprises maps of Sen’s slopes and p-values. These outputs can be used to determine which pixels in the multidimensional time series have a statistically significant (i.e., less than 0.05 significance level) monotonic trend.

The seasonal-Kendall test^46–48^ is a nonparametric statistical test used to detect monotonic trends in time series data that exhibit a seasonal pattern. It is an extension of the Kendall rank correlation test, which measures the strength of association between two variables by assessing the degree of similarity between the ranks of the corresponding values. The seasonal-Kendall test considers the seasonal nature of the data by dividing the time series into seasonal periods and testing for trends within each period. To conduct the test, the time series data is first divided into seasons of equal length and then the test is applied to each season separately. The test is based on the number of concordant and discordant pairs of observations in each season. A concordant pair is one in which the values of two observations move in the same direction over time, while a discordant pair is one in which the values move in opposite directions. The test statistic is then calculated based on the difference between the number of concordant and discordant pairs. Assuming that the trend is linear, Sen’s slope^49^, which is also a nonparametric measure of the magnitude and direction of a monotonic trend in time series data, is then calculated. It is computed as the median of the slopes between all pairs of data points in the time series.

Finally, the most vulnerable areas in the Arab world to increasing air temperatures and decreasing precipitation amounts were identified by applying raster-based overlay analysis (https://pro.arcgis.com/en/pro-app/latest/tool-reference/spatial-analyst/understanding-overlay-analysis.htm). These areas should have statistically significant increasing monotonic trends of maximum and minimum air temperature and statistically significant monotonic decreasing trends of precipitation amount. First, maps of Sen’s slope of maximum air temperature, minimum air temperature, and precipitation amount were reclassified using the ‘Reclassify’ tool. Pixels with statistically significant positive Sen’s slope of maximum air temperature and minimum air temperature and statistically significant negative Sen’s Slope of precipitation amount were assigned the value of 1. Then, the reclassified rasters were added using the ‘Raster Calculator’ tool. Pixels in the resulted output map with values of 3 represent the most vulnerable areas in the Arab world to increasing air temperatures and decreasing precipitation amounts.

## Results and discussion

### Spatial and temporal patterns of air temperature

**Figure 2:**
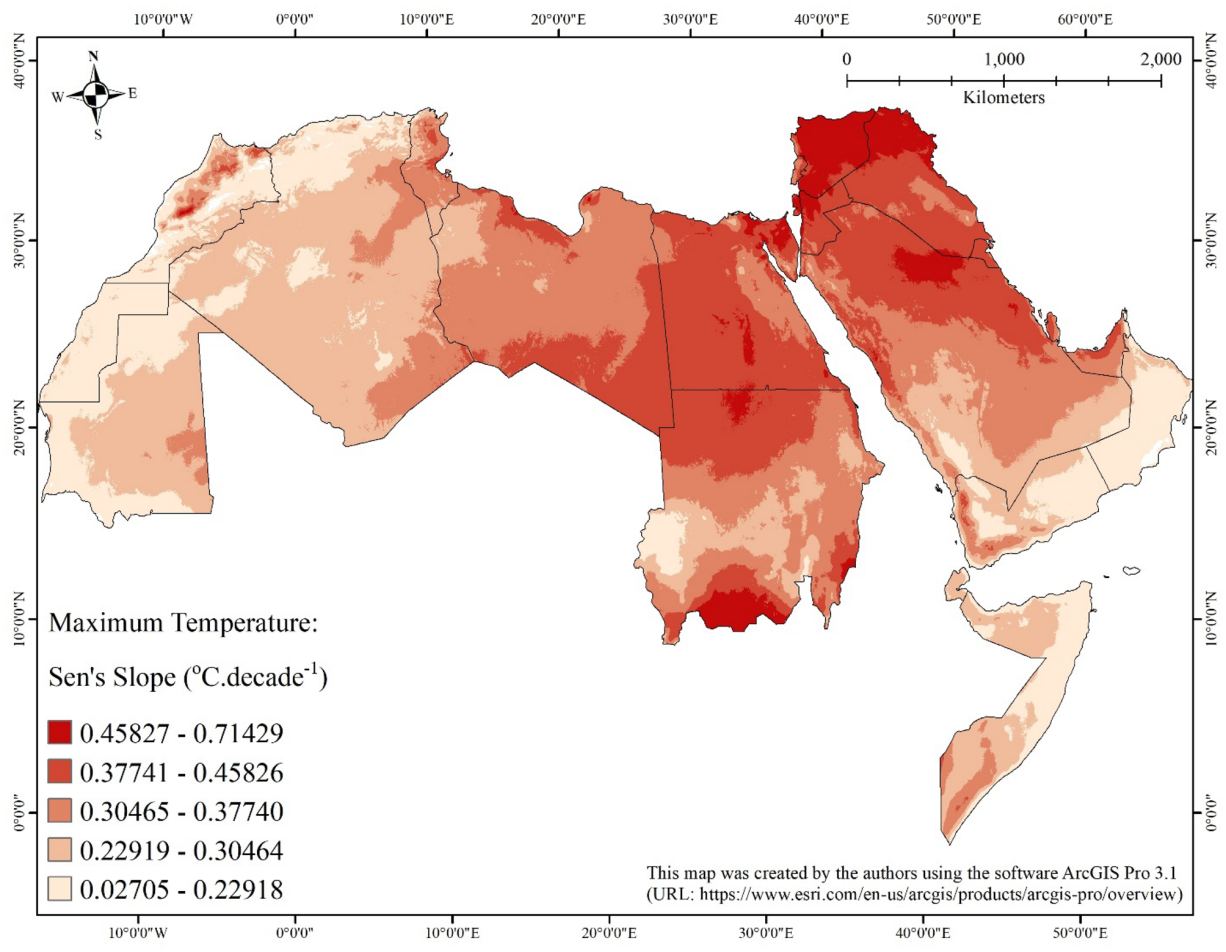
Map showing statistically significant (at 0.05 significance level) Sen’s slopes of the seasonal Kendall trend analysis applied on maximum temperature in the Arab world from January 1980 to December 2018.

**Figure 3:**
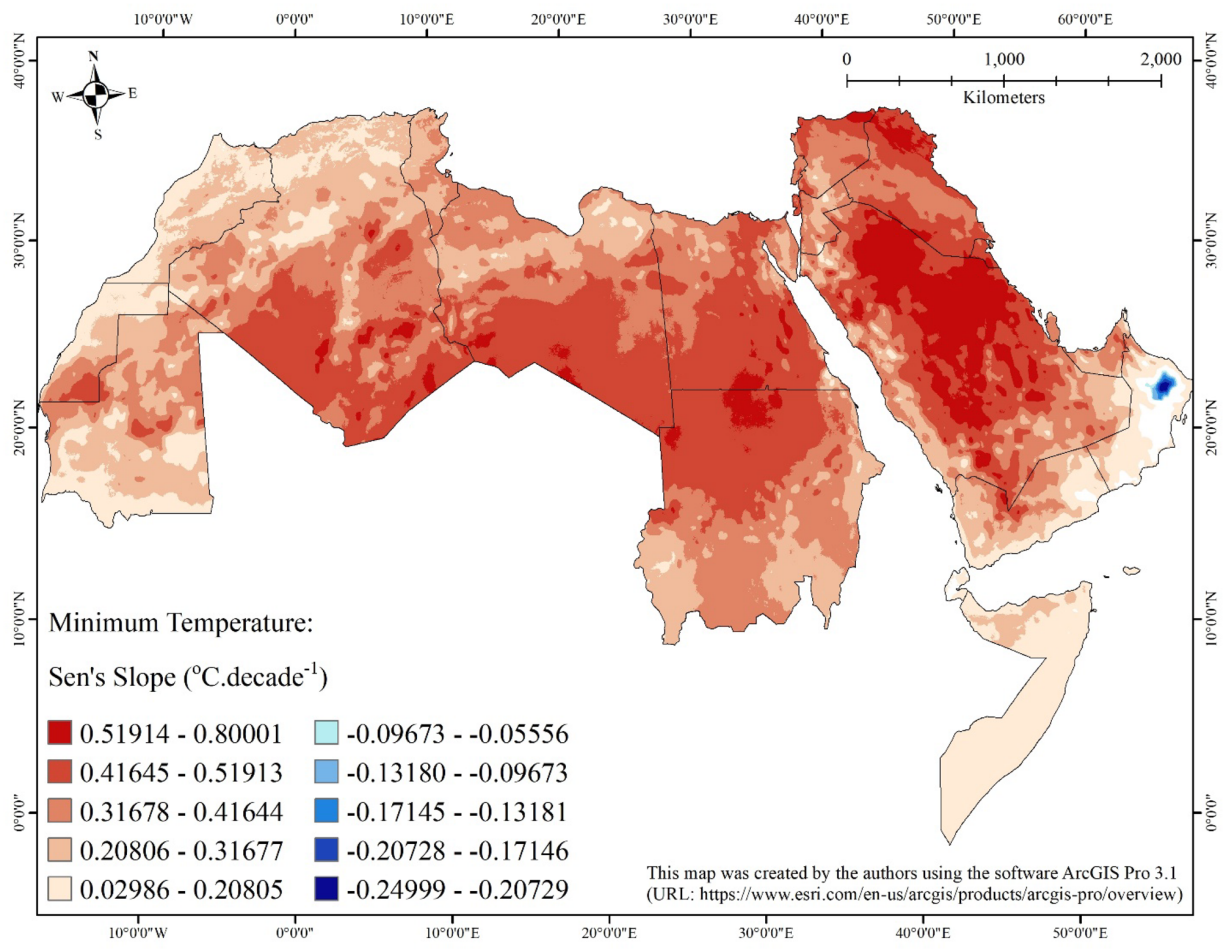
Map showing statistically significant (at 0.05 significance level) Sen’s slopes of the seasonal Kendall trend analysis applied on minimum temperature in the Arab world from January 1980 to December 2018.

Almost all areas of the Arab world (99%) show statistically significant positive monotonic trends (i.e., a general increase over time) of either maximum air temperature (Table 1; Fig. 2) or minimum air temperature (Table 2; Fig. 3). Less than 1% of the areas of the Arab world did not show statistically significant negative nor positive monotonic trends of either maximum air temperature or minimum air temperature.

**Table 1:** Summary statistics of statistically significant (at 0.05 significance level) Sen’s slopes of the seasonal Kendall trend analysis applied on monthly maximum temperature in the Arab world from January 1980 to December 2018.

Country	Statistically significant negative slope (°C decade^−1^)						Statistically significant positive slope (°C decade^−1^)						Remaining area (km^2^)
	Area (km^2^)	Minimum	Maximum	Mean	Standard deviation	Median	Area (km^2^)	Minimum	Maximum	Mean	Standard deviation	Median	
Algeria	0	–	–	–	–	–	2,318,664	0.10527	0.40002	0.26562	0.04204	0.26668	7161
Bahrain	0	–	–	–	–	–	628	0.27272	0.34699	0.28847	0.01269	0.28572	0
Djibouti	0	–	–	–	–	–	21,495	0.13158	0.29999	0.24462	0.02749	0.25000	0
Egypt	0	–	–	–	–	–	1,002,126	0.24999	0.51724	0.41051	0.03479	0.41667	0
Iraq	0	–	–	–	–	–	437,271	0.27272	0.66666	0.43218	0.05047	0.42308	0
Jordan	0	–	–	–	–	–	89,462	0.33333	0.66667	0.43208	0.04168	0.42106	0
Kuwait	0	–	–	–	–	–	16,774	0.28573	0.47368	0.42332	0.03133	0.43749	0
Lebanon	0	–	–	–	–	–	10,218	0.30769	0.54545	0.38853	0.04257	0.38095	0
Libya	0	–	–	–	–	–	1,623,602	0.20639	0.50000	0.35615	0.03821	0.35714	0
Mauritania	0	–	–	–	–	–	1,041,566	0.07143	0.36363	0.23885	0.04110	0.24996	1888
Morocco	0	–	–	–	–	–	380,578	0.07408	0.50000	0.23619	0.06477	0.22224	23,510
Oman	0	–	–	–	–	–	298,770	0.04347	0.36363	0.17718	0.04812	0.18182	11,312
Gaza Strip	0	–	–	–	–	–	358	0.39695	0.47060	0.43424	0.02143	0.44000	0
West Bank	0	–	–	–	–	–	5856	0.38888	0.58334	0.47821	0.03916	0.47913	0
Qatar	0	–	–	–	–	–	11,096	0.28000	0.42860	0.37205	0.03568	0.38095	0
Saudi Arabia	0	–	–	–	–	–	1,962,879	0.14815	0.50000	0.35136	0.06488	0.35001	0
Somalia	0	–	–	–	–	–	639,845	0.05554	0.43478	0.23745	0.05891	0.24242	0
Sudan	0	–	–	–	–	–	1,865,167	0.07142	0.61904	0.35517	0.07466	0.35000	0
Syria	0	–	–	–	–	–	188,317	0.31250	0.71429	0.51569	0.05987	0.50001	0
Tunisia	0	–	–	–	–	–	155,504	0.12498	0.46154	0.32145	0.04368	0.33331	110
UAE	0	–	–	–	–	–	70,548	0.10344	0.45000	0.35093	0.04961	0.35714	0
Western Sahara	0	–	–	–	–	–	268,916	0.06248	0.24998	0.20137	0.01982	0.20001	1330
Yemen	0	–	–	–	–	–	422,734	0.02705	0.45308	0.23362	0.05582	0.22727	3156

**Table 2:** Summary statistics of statistically significant (at 0.05 significance level) Sen’s slopes of the seasonal Kendall trend analysis applied on monthly minimum temperature in the Arab world from January 1980 to December 2018.

Country	Statistically significant negative slope (°C decade^−1^)						Statistically significant positive slope (°C decade^−1^)						Remaining area (km^2^)
	Area (km^2^)	Minimum	Maximum	Mean	Standard deviation	Median	Area (km^2^)	Minimum	Maximum	Mean	Standard deviation	Median	
Algeria	0	–	–	–	–	–	2,325,825	0.09091	0.66667	0.35389	0.10175	0.36666	0
Bahrain	0	–	–	–	–	–	628	0.28571	0.33333	0.29866	0.00999	0.29630	0
Djibouti	0	–	–	–	–	–	12,240	0.03334	0.16668	0.09189	0.02725	0.09091	9255
Egypt	0	–	–	–	–	–	1,002,126	0.11538	0.66664	0.42116	0.06716	0.43333	0
Iraq	0	–	–	–	–	–	437,271	0.30496	0.62500	0.44130	0.05858	0.43614	0
Jordan	0	–	–	–	–	–	89,462	0.14287	0.50000	0.32638	0.05842	0.33333	0
Kuwait	0	–	–	–	–	–	16,774	0.33325	0.56250	0.47397	0.04767	0.49998	0
Lebanon	0	–	–	–	–	–	10,218	0.30434	0.45455	0.37517	0.03164	0.37500	0
Libya	0	–	–	–	–	–	1,623,602	0.15790	0.59998	0.39465	0.08295	0.40909	0
Mauritania	0	–	–	–	–	–	1,043,266	0.06250	0.50000	0.26804	0.07630	0.26316	188
Morocco	0	–	–	–	–	–	402,934	0.04762	0.38462	0.21423	0.04913	0.20690	1154
Oman	21,026	-0.24999	-0.05556	-0.13971	0.04901	-0.13332	193,770	0.03226	0.46153	0.15768	0.08065	0.12904	95,286
Gaza Strip	0	–	–	–	–	–	358	0.35714	0.43333	0.40417	0.02458	0.41380	0
West Bank	0	–	–	–	–	–	5856	0.38462	0.49998	0.43107	0.01820	0.42858	0
Qatar	0	–	–	–	–	–	11,096	0.27778	0.41667	0.35846	0.03032	0.36363	0
Saudi Arabia	0	–	–	–	–	–	1,962,879	0.11110	0.80001	0.45580	0.11839	0.47619	0
Somalia	0	–	–	–	–	–	639,129	0.03176	0.30556	0.13116	0.04890	0.11539	716
Sudan	0	–	–	–	–	–	1,865,167	0.13793	0.56251	0.38356	0.08576	0.37037	0
Syria	0	–	–	–	–	–	188,317	0.18983	0.64402	0.39751	0.07448	0.39999	0
Tunisia	0	–	–	–	–	–	155,614	0.15790	0.40683	0.30409	0.03710	0.30769	0
UAE	0	–	–	–	–	–	70,548	0.11483	0.46154	0.31355	0.06403	0.32353	0
Western Sahara	0	–	–	–	–	–	269,982	0.04084	0.50000	0.26124	0.09776	0.21739	264
Yemen	0	–	–	–	–	–	417,542	0.02985	0.56386	0.25586	0.09490	0.25002	8348

Regarding maximum air temperature, the magnitude of the increase varies from 0.027 °C/decade (in Yemen) to 0.714 °C/decade (in Syria) with a mean of 0.318 °C/decade. On country-by-country basis, the largest mean of magnitude of increase was calculated for Syria (0.516 °C/decade), followed by Palestine (0.478 °C/decade for West Bank and 0.434 °C/decade for Gaza Strip), and then Iraq (0.432 °C/decade); while the lowest was calculated for Oman (0.177 °C/decade), followed by Western Sahara (0.201 °C/decade), and then Yemen (0.234 °C/decade).

With respect to minimum air temperature, the magnitude of the increase varies from 0.030 °C/decade (in Yemen) to 0.800 °C/decade (in Saudi Arabia) with a mean of 0.356 °C/decade. On country-by-country basis, the largest mean of magnitude of increase was calculated for Kuwait (0.474 °C/decade), followed by Saudi Arabia (0.456 °C/decade), and then Iraq (0.441 °C/decade); while the lowest was calculated for Djibouti (0.092 °C/decade), followed by Somalia (0.131 °C/decade), and then Oman (0.158 °C/decade). Small areas (0.2%) in Oman, namely the areas surrounding the Hajar Mountains in the northern parts of the country, show statistically significant negative monotonic trends (i.e., decrease over time) of minimum air temperature with magnitudes varying from −0.056 to −0.250 °C/decade, and a mean of −0.140 °C/decade.

The largest magnitude of increase of maximum air temperature (greater than 0.5 °C/decade) covers northern Iraq, almost all of Syria, entirety of Palestine, northwestern parts of Jordan, and northern Sinai Peninsula, in addition to scattered patches in northern Saudi Arabia along the borders with Iraq and Kuwait, some parts of the Nile River Delta, southern parts of Sudan, the deserts in southern Egypt and northern Sudan, and Atlas Mountains in Morocco. The spatial pattern of the largest magnitude of increasing trend of minimum air temperature (greater than 0.5 °C/decade) is more scattered with small patches distributed all over different parts of the Arab world, except Oman, Somalia, and northwestern Africa (Tunisia, northern Algeria, and Morocco). Maximum and minimum air temperature increasing trends and rates for Southern Levant, which encompasses Palestine, Jordan, and southern Syria go along with previously published findings^50,51^.

The observed increase in minimum and maximum temperatures match corresponding trends from other parts of the globe. However, the rates of increase tend to be higher for land-locked areas, that is, those located far from main water bodies. This is likely attributed to the fact that most heat goes to oceans and seas because the specific heat of water is higher than land. Coastal areas do not exhibit similar temperature rise because most heat goes into main water bodies since water has higher values of specific heat than land^52^.

### Spatial and temporal patterns of precipitation

**Figure 4:**
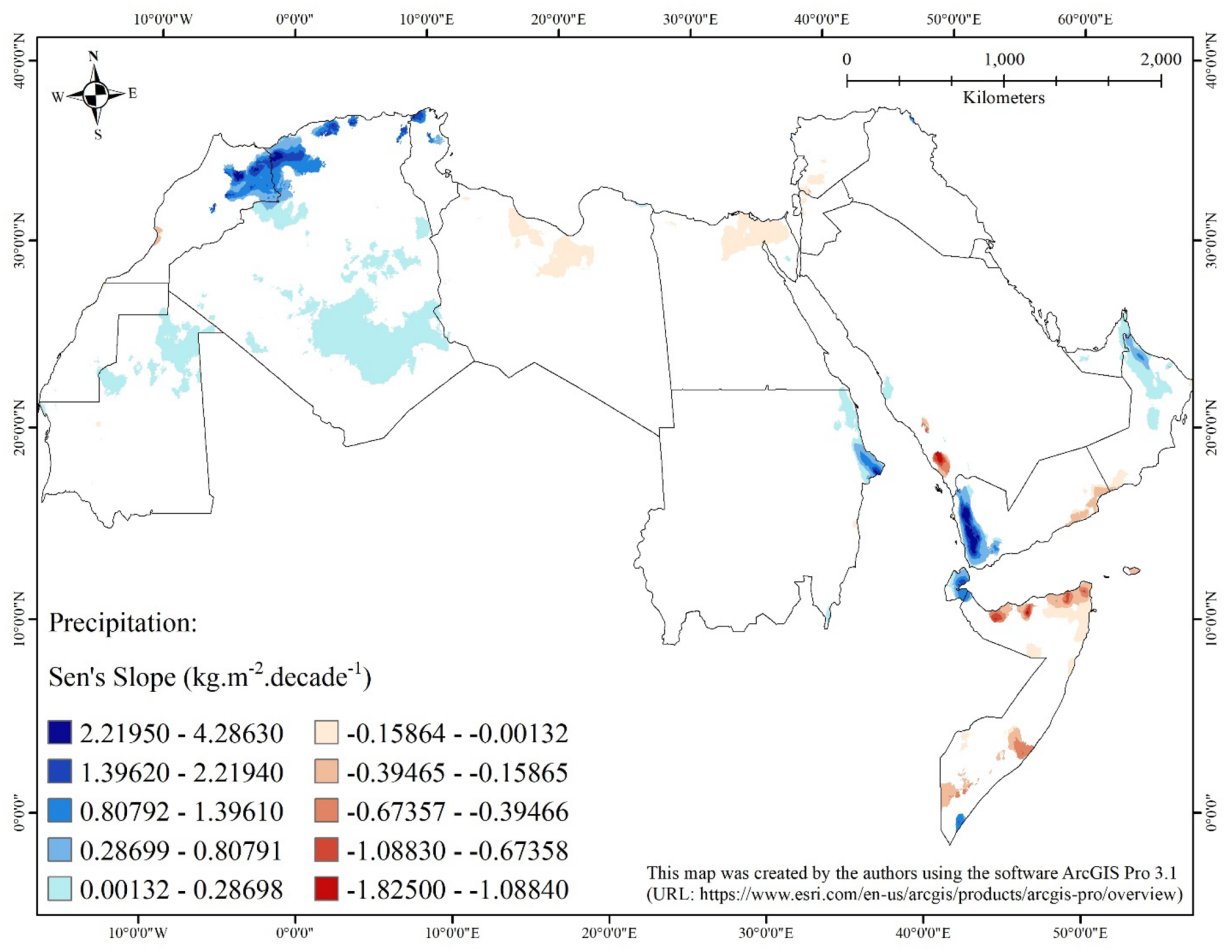
Map showing statistically significant (at 0.05 significance level) Sen’s slopes of the seasonal Kendall trend analysis applied on precipitation in the Arab world from January 1980 to December 2018.

Analysis of the spatial and temporal trends of precipitation amounts in the Arab world (Table 3; Fig. 4) allows it to be classified into three regions. The first are areas that show statistically significant negative monotonic trends (i.e., generally, a decrease in precipitation over time). The second are areas that show statistically significant positive monotonic trends (i.e., generally, an increase in precipitation over time). The third are areas in which no statistically significant negative nor positive monotonic trends (i.e., no change in precipitation) can be discerned.

**Table 3:** Summary statistics of statistically significant (at 0.05 significance level) Sen’s slopes of the seasonal Kendall trend analysis applied on monthly precipitation in the Arab world from January 1980 to December 2018.

Country	Statistically significant negative slope (kg m^−2^ decade^−1^)						Statistically significant positive slope (kg m^−2^ decade^−1^)						Remaining area (km^2^)
	Area (km^2^)	Minimum	Maximum	Mean	Standard deviation	Median	Area (km^2^)	Minimum	Maximum	Mean	Standard deviation	Median	
Algeria	0	–	–	–	–	–	484,487	0.00132	3.88619	0.23745	0.46940	0.02857	1,841,338
Bahrain	0	–	–	–	–	–	0	–	–	–	–	–	628
Djibouti	0	–	–	–	–	–	16,462	0.10000	1.98833	0.73825	0.46058	0.65971	5033
Egypt	70,062	−0.07500	−0.00132	−0.02719	0.01247	−0.02500	1315	0.00132	0.01818	0.00707	0.00352	0.00635	930,749
Iraq	0	–	–	–	–	–	852	0.38918	1.37275	0.81707	0.21168	0.81583	436,419
Jordan	2988	−0.15436	−0.00714	−0.06677	0.02700	−0.06069	0	–	–	–	–	–	86,474
Kuwait	0	–	–	–	–	–	0	–	–	–	–	–	16,774
Lebanon	0	–	–	–	–	–	0	–	–	–	–	–	10,218
Libya	76,822	−0.10556	−0.00132	−0.03168	0.02289	−0.02143	5394	0.00132	0.14772	0.01516	0.01960	0.00625	1,541,386
Mauritania	943	−0.02500	−0.00385	−0.01771	0.00562	−0.02000	106,721	0.00132	0.02500	0.01081	0.00458	0.01053	935,790
Morocco	4050	−0.37297	−0.04464	−0.23402	0.07063	−0.24614	72,317	0.09403	4.10313	1.11337	0.50488	1.05000	327,721
Oman	8859	−0.24734	−0.00435	−0.07121	0.04680	−0.06667	59,980	0.00132	1.01381	0.14124	0.19960	0.03539	241,244
Gaza Strip	0	–	–	–	–	–	0	–	–	–	–	–	358
West Bank	0	–	–	–	–	–	0	–	–	–	–	–	5856
Qatar	0	–	–	–	–	–	0	–	–	–	–	–	11,096
Saudi Arabia	15,223	−1.82500	−0.05670	−0.60937	0.38542	−0.52614	6725	0.00132	0.94488	0.05557	0.06430	0.03333	1,940,932
Somalia	132,699	−1.27802	−0.00132	−0.27265	0.21345	−0.24375	6931	0.14514	1.43964	0.98673	0.27172	1.03026	500,215
Sudan	1859	−0.01000	−0.00132	−0.00525	0.00167	−0.00500	50,950	0.00132	2.64562	0.35144	0.46297	0.15820	1,812,358
Syria	9359	−0.19137	−0.00132	−0.04551	0.04247	−0.03077	187	0.49069	1.17712	0.89838	0.16378	0.95359	178,771
Tunisia	0	–	–	–	–	–	9639	0.00833	2.16865	1.13862	0.40196	1.14981	145,975
UAE	0	–	–	–	–	–	11,188	0.00143	0.48889	0.09700	0.09347	0.06364	59,360
Western Sahara	1041	−0.09167	−0.04211	−0.06608	0.00822	−0.06667	3585	0.00556	0.02500	0.01588	0.00340	0.01550	265,620
Yemen	23,358	−0.40000	−0.00139	−0.18955	0.06530	−0.19069	64,080	0.00909	4.28634	1.15318	0.91654	0.78269	338,451

We found that most of the areas of the Arab world (90%) belong to the third type of region where no trends can be observed. Most of these areas (69%) exist in Saudi Arabia (15%), Algeria (14%), Sudan (14%), Libya (12%), Mauritania (7%), and Egypt (7%). The entirety of five countries of the Arab world also belongs to this region, they are: Qatar, Bahrain, Kuwait, Lebanon, and Palestine. The remaining 10% of the areas of the Arab world show either increasing or decreasing trends.

About 7% of the area of the Arab world belongs to the second type of region with increase in precipitation over time. More than half of the areas with increased precipitation can be observed in Algeria (4%). The magnitude of the increase varies from 0.001 kg m^−2^/decade (in Mauritania, Algeria, Libya, Egypt, Sudan, Saudi Arabia, and Oman) to 4.286 kg m^−2^/decade (in Yemen), with a mean of 0.366 kg m^−2^/decade. The regions with largest increase (greater than 1.0 kg m^−2^/decade) can be observed mainly along the high mountain ranges in the Arab world. These mountain ranges include the Atlas Mountains in the far west of the Arab world (in Morocco, Algeria, and Tunisia), the southern banks of the Red Sea along the Yemen-Hadhramout Mountains in Yemen, the mountains in Djibouti and northeastern Sudan, the Hajar Mountains in northern Oman, and the high mountain ridges of northeastern Iraqi Kurdistan. Increases of less than 1.0 kg m^−2^/decade can be noticed mainly in the African Sahara in central Algeria (Hoggar Mountain Ranges), northern Mauritania, northeastern Sudan along the Red Sea coast, on the opposite side of the sea in central Asir Mountains in western Saudi Arabia, in addition to northeastern Oman and northeastern United Arab Emirates along the Gulf of Oman. On country-by-country basis, the largest mean of magnitude of increase was obtained for Yemen (1.153 kg m^−2^/decade), followed by Tunisia (1.139 kg m^−2^/decade), and then Morocco (1.113 kg m^−2^/decade); while the lowest was obtained for Egypt (0.007 kg m^−2^/decade), followed by Mauritania (0.011 kg m^−2^/decade), and then Libya (0.015 kg m^−2^/decade).

About 3% of the area of the Arab world belongs to the first type of region, with decreasing amounts of precipitation. Most of the areas with decrease can be noticed in Somalia (1%). The magnitude of the decrease varies from −0.001 kg m^−2^/decade (in Libya, Egypt, Sudan, Somalia, and Syria) to −1.825 kg m^−2^/decade (in Saudi Arabia) with a mean of −0.163 kg m^−2^/decade. The largest magnitudes of decrease (less than −0.4 kg m^−2^/decade) can be observed mainly as scattered patches in Somalia, specifically along the coastline of the Gulf of Aden, on the opposite side of the gulf in southeastern Yemen, and in southern Asir mountains in southwestern Saudi Arabia. Decreases in precipitation greater than −0.4 kg m^−2^/decade can be observed mainly in northern Libya (along the Mediterranean Sea), northern Egypt (including some parts of the Nile River Delta and northern Sinai Peninsula along the Mediterranean Sea), southwestern Syria and northwestern Jordan (in addition to the eastern bank of the Dead Sea), with scattered patches occurring mainly in northern Somalia, southeastern Yemen, and southwestern Oman. On country-by-country basis, the largest mean of magnitude of decrease was calculated for Saudi Arabia (−0.609 kg m^−2^/decade), followed by Somalia (−0.273 kg m^−2^/decade), and then Morocco (−0.234 kg m^−2^/decade); while the lowest was calculated for Sudan (−0.005 kg m^−2^/decade), followed by Mauritania (−0.018 kg m^−2^/decade), and then Egypt (−0.027 kg m^−2^/decade). Decreasing precipitation in some Arabian territories have been reported by Salameh et al. (2022)^53^ who detected declining precipitation trends in spring and autumn.

It is evident from these results that most of the Arab world does not exhibit clear increase or decrease in precipitation. Few exceptions located in the western Mediterranean (i.e., Morocco and Algeria) demonstrate increasing trends in precipitation. Other exceptions in the eastern Mediterranean territories (i.e., in some parts of Libya, Egypt and Syria) exhibit decreasing precipitation trends. This goes along with the findings of Cook et al. (2015)^5^ who detected a “*SEASAW*” precipitation trend in the West- and East-Mediterranean.

### Areas of most vulnerability and potential impacts

**Figure 5:**
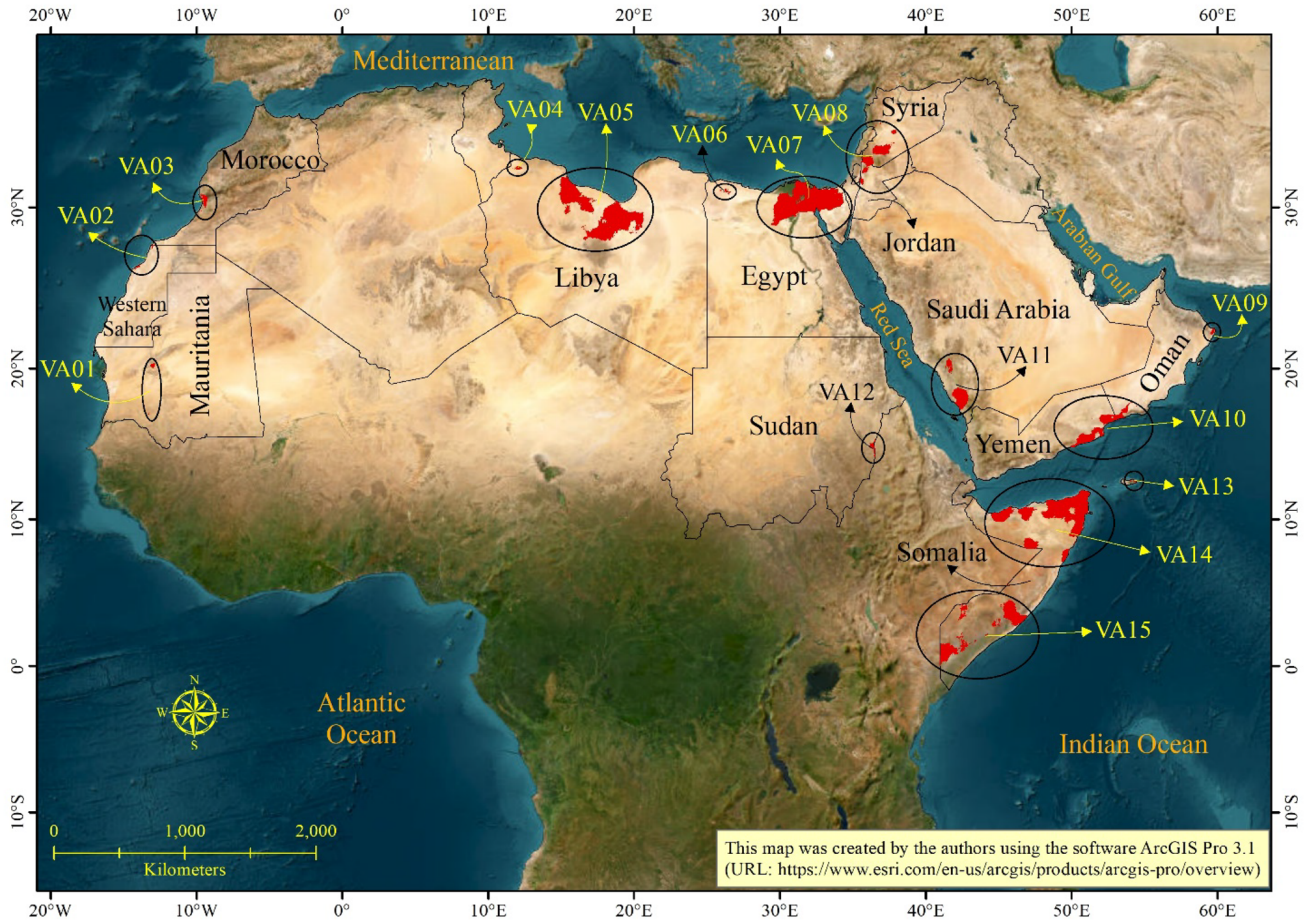
Map showing regions of heightened vulnerability in the Arab world (depicted as solid red polygons). These areas are grouped arbitrarily into neighboring clusters, enclosed by ovals of varying sizes. The designations VA01 to VA15 correspond to vulnerable areas 1 through 15.

Overlay analysis (Table 4; Fig. 5) identified multiple areas in the Arab world as the most vulnerable to increasing trends of maximum and minimum air temperatures and decreasing trends of precipitation amounts. These areas are scattered in various locations in Mauritania, Western Sahara, Morocco, Libya, Egypt, Jordan, Syria, Oman, Yemen, Saudi Arabia, Sudan, and Somalia with a total area of 336,694.6 km^2^.

**Table 4:** List of the most vulnerable areas in the Arab world.

Vulnerable area code	Area (km^2^)	Vulnerable area description
VA01	943.4	Unpopulated areas in the southern and central parts of Mauritania
VA02	648.5	Unpopulated areas in northwest of Western Sahara and at the borders between Western Sahara and Morocco on the shores of the Atlantic Ocean
VA03	2201.9	Includes Agadir City along Morocco’s southern Atlantic coast
VA04	745.7	Unpopulated area in the northwestern parts of Libya close to the Tunisian Borders
VA05	76,076.5	Unpopulated area in the northern parts of Libya on the shores of the Mediterranean
VA06	755.7	Unpopulated area in the northwestern parts of Egypt
VA07	69,348.6	Includes Cairo City and its suburbs and extends to the Nile River Delta on the shores of the Mediterranean in addition to the unpopulated area in the northern parts of Sinai Desert
VA08	12,338.1	Includes the northeastern parts of the Dead Sea and northwestern parts of Jordan (i.e., northern parts of Amman City, Salt City, Jerash City, and Irbid City and its suburbs) Also, includes the southwestern parts of Syria (i.e., eastern shores of Lake Tiberias and Daraa City and its suburbs) and southeastern parts of Damascus City, Homs City, and an unpopulated area in central Syria
VA09	827.8	Unpopulated area in the eastern parts of Oman on the shores of the Arabian Sea
VA10	23,014.0	Unpopulated area at the borders between Oman and Yemen on the shores of the Arabian Sea
VA11	15,222.8	Includes the cities of Abha, Khamis Mushait, and Al Baha in the southwestern parts of Saudi Arabia in Asir area on the shores of the Red Sea
VA12	1859.2	Unpopulated area in the eastern parts of Sudan
VA13	336.8	Unpopulated area in the eastern parts of Socotra Island of Yemen in the Gulf of Aden
VA14	90,145.6	Includes unpopulated areas in addition to low density settlements in the African Horn in the northern parts of Somalia
VA15	42,230.0	Includes unpopulated areas in addition to low density settlements in the southern parts of Somalia
Total	336,694.6 km^2^

Carefully studying these areas, we found that nine of these areas exist in locations where no human populations are present (i.e., VA01, VA02, VA04, VA05, VA06, VA09, VA10, VA12, and VA13). Further, two of the vulnerable areas (i.e., VA14 and VA15) exist in northern and southern Somalia. In these areas, only low-density scattered settlements exist, if at all. Most of these areas are devoid of human population. The remaining four vulnerable areas cover several major urban centers and their surroundings in the Arab world. These urban centers are Agadir in Morocco (VA03); Cairo and Nile River Delta in Egypt (VA07); northeast Dead Sea, north Amman, Salt, Jerash, and Irbid in Jordan (VA08); east Lake Tiberias, Daraa, southeast Damascus, and Homs in Syria (VA08); and Abha, Khamis Mushait, and Al Baha in Saudi Arabia (VA11).

This clear and rapid temperature rise accompanied by lack of precipitation will inevitably lead to an increase in energy demand for cooling and water desalination, as a consequence of anticipated drought. A set of other challenges would also put extra pressure on freshwater demand and air conditioning, including urbanization and rapid population growth, thereby exerting stress on old energy infrastructure in most Arab countries. This will necessitate the construction of additional power plants resulting in direct and indirect emissions of greenhouse gases creating a positive feedback loop.

Several parts of the Arab World including the great Sahara, Jordan, Saudi Arabia, and Gulf states are already in a state of severe water stress due to the harsh climatic conditions that dominate the region as it is influenced by persisting high pressure ridges. Therefore, these territories contribute small portions to the water budget in the region. Communities in these areas rely on ground water resources for agriculture or domestic uses. Wealthy states such as Saudi Arabia and United Arab Emirates fulfill most of their domestic water needs via sea water desalination (energy intensive), but countries like Jordan, Lebanon, Palestine, Somalia, Mauretania, and Yemen lack local energy sources, which puts them in water jeopardy. Therefore, strategic water plans in these countries must cautiously deal with unreliable and unpredicted precipitation trends.

### Factors influence climate in the Arab world

Most countries in the Arab world are influenced by synoptic meteorological factors and large-scale atmospheric patterns that play a role in shaping the region's climate and can contribute to changes in temperature, precipitation patterns, and other climate-related phenomena^54^. They include the subtropical high-pressure systems, Mediterranean Sea influence, north Atlantic oscillation (NAO), Mediterranean oscillation, east Atlantic/west Russia pattern, Saharan air layer, jet streams, tropical and subtropical cyclones, monsoonal flows. As climate change progresses, some of these synoptic meteorological factors may be influenced by shifts in atmospheric circulation and temperature patterns. Changes in sea surface temperatures and atmospheric pressure systems can contribute to alterations in regional climate.

Current climatic changes around the glob are largely blamed on global warming which is caused by the accumulation of greenhouse gases in the atmosphere, primarily from human activities such as burning fossil fuels (coal, oil, and natural gas), deforestation, and industrial processes^55^. While the impacts of global warming can vary based on geographic location, socioeconomic factors, and regional policies, many Arab countries may be particularly susceptible to its effects because they exist in regions characterized by arid or semi-arid climates, such as the MENA region. These regions are already prone to high temperatures, limited freshwater resources, and drought. Global warming exacerbates these conditions, leading to increased heatwaves, water scarcity, and desertification. Countries in this region often face water scarcity issues due to their arid climates. Increased temperatures can cause faster evaporation rates from water bodies and soil, leading to further water shortages.

Some Arab countries face political instability and conflict, which can hinder their ability to address the impacts of global warming effectively^55^. Additionally, socioeconomic factors may impact a country's capacity to invest in climate adaptation and mitigation strategies. Several countries in the region lack the infrastructure and resources necessary to adapt to cope with the climate change. This can include inadequate water management systems, insufficient disaster preparedness, and limited access to technology for monitoring and mitigating climate-related risks.

## Conclusions

The study yielded several key findings regarding climate indicators trends in the Arab world over the past four decades:

1. Maximum Air Temperature Patterns: Nearly all areas in the Arab world experienced statistically significant increasing trends in maximum air temperatures over time. The magnitude of this increase varied across the region, with Yemen showing the smallest increase at 0.027 °C/decade, while Syria had the largest increase at 0.714 °C/decade, with a mean increase of 0.318 °C/decade. Syria had the highest average increase (0.516 °C/decade), while Oman had the lowest (0.177 °C/decade).
2. Minimum Air Temperature Patterns: Similarly, nearly all areas in the Arab world exhibited statistically significant increasing trends in minimum air temperatures over time. The magnitude of increase also varied, ranging from 0.030 °C/decade in Yemen to 0.800 °C/decade in Saudi Arabia, with a mean increase of 0.356 °C/decade. Kuwait had the highest average increase (0.474 °C/decade), while Djibouti had the lowest (0.092 °C/decade). Notably, certain areas around Oman’s Hajar Mountains showed statistically significant decreasing trends in minimum air temperatures, ranging from −0.056 to −0.250 °C/decade, with a mean decrease of −0.140 °C/decade.
3. Precipitation Patterns: The majority of the Arab world (90%) did not exhibit statistically significant trends in precipitation over the study period. However, around 7% of the region experienced statistically significant increasing trends in precipitation. These increases varied across the Arab world, with Yemen showing the highest increase at 4.286 kg m^−2^/decade, and Mauritania, Algeria, Libya, Egypt, Sudan, Saudi Arabia, and Oman showing the lowest increase at 0.001 kg m^−2^/decade, with a mean increase of 0.366 kg m^−2^/decade. Yemen had the highest average increase (1.153 kg m^−2^/decade), while Egypt had the lowest (0.007 kg m^−2^/decade). Approximately 3% of the Arab world faced statistically significant decreasing trends in precipitation. These decreases also varied across the Arab world, with Saudi Arabia experiencing the highest decrease at −1.825 kg m^−2^/decade, and Libya, Egypt, Sudan, Somalia, and Syria experiencing the lowest decrease at −0.001 kg m^−2^/decade, with a mean decrease of −0.163 kg m^−2^/decade. Saudi Arabia had the highest average decrease (−0.609 kg m^−2^/decade), while Sudan the lowest (−0.005 kg m^−2^/decade).
4. Vulnerable Areas: Vulnerable areas in the Arab world, characterized by increasing maximum and minimum air temperatures and decreasing precipitation trends, were identified in Mauritania, Western Sahara, Morocco, Libya, Egypt, Jordan, Syria, Oman, Yemen, Saudi Arabia, Sudan, and Somalia. These areas covered a total of 336,694.6 km^2^ and included major urban centers and their surroundings in Agadir (Morocco), Cairo and the Nile River Delta (Egypt), northeast Dead Sea, north Amman, Salt, Jerash, and Irbid (Jordan), east Lake Tiberias, Daraa, southeast Damascus, and Homs (Syria), and Abha, Khamis Mushait, and Al Baha (Saudi Arabia).

## Data Availability

All the data are derived from the following source available in the public domain: [https://chelsa-climate.org/].
